# Ethical issues when using digital biomarkers and artificial intelligence for the early detection of dementia

**DOI:** 10.1002/widm.1492

**Published:** 2023-02-19

**Authors:** Elizabeth Ford, Richard Milne, Keegan Curlewis

**Affiliations:** ^1^ Department of Primary Care and Public Health Brighton and Sussex Medical School Brighton UK; ^2^ Kavli Centre for Ethics, Science and the Public University of Cambridge Cambridge UK; ^3^ Engagement and Society Wellcome Connecting Science Cambridge UK; ^4^ Royal Free London NHS Foundation Trust London UK

**Keywords:** artificial intelligence, dementia, digital biomarkers, early detection, ethics

## Abstract

Dementia poses a growing challenge for health services but remains stigmatized and under‐recognized. Digital technologies to aid the earlier detection of dementia are approaching market. These include traditional cognitive screening tools presented on mobile devices, smartphone native applications, passive data collection from wearable, in‐home and in‐car sensors, as well as machine learning techniques applied to clinic and imaging data. It has been suggested that earlier detection and diagnosis may help patients plan for their future, achieve a better quality of life, and access clinical trials and possible future disease modifying treatments. In this review, we explore whether digital tools for the early detection of dementia can or should be deployed, by assessing them against the principles of ethical screening programs. We conclude that while the importance of dementia as a health problem is unquestionable, significant challenges remain. There is no available treatment which improves the prognosis of diagnosed disease. Progression from early‐stage disease to dementia is neither given nor currently predictable. Available technologies are generally not both minimally invasive and highly accurate. Digital deployment risks exacerbating health inequalities due to biased training data and inequity in digital access. Finally, the acceptability of early dementia detection is not established, and resources would be needed to ensure follow‐up and support for those flagged by any new system. We conclude that early dementia detection deployed at scale via digital technologies does not meet standards for a screening program and we offer recommendations for moving toward an ethical mode of implementation.

This article is categorized under:

Application Areas > Health Care
Commercial, Legal, and Ethical Issues > Ethical Considerations
Technologies > Artificial Intelligence

## Graphical abstract

The early detection of dementia poses many quandaries for clinicians and potential harm for patients, as well as possible benefits.

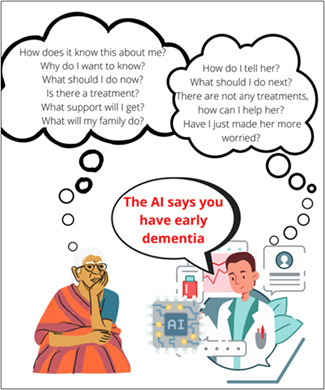

## 1 INTRODUCTION

Dementia has been identified as a key potential area of application for the development of digital biomarkers and automated tools. However, the development and implementation of these tools at scale also raises a range of ethical questions. In this review, we set out the context of dementia and the scope for early detection, and review some of these ethical questions through the lens of population screening.

### 1.1 What is dementia?

Dementia is a term referring to a range of disorders characterized by a progressive decline in memory, reasoning, communication, and the ability to carry out everyday activities. These changes are usually associated with progressive structural and chemical changes which damage regions of the brain (Banerjee, 2010). The most common type of dementia is Alzheimer's disease (60%–70% of cases) followed by vascular dementia (20%) and dementia with Lewy Bodies (15%; Alzheimer's Research U. K. Dementia Statistics Hub, 2018). Currently there are no treatments which are curative for dementia, however, a timely diagnosis of dementia can still be valuable particularly for younger patients and their families, as they can be offered a range of supportive or therapeutic interventions, inform themselves about the condition, and plan for their future financial and care needs, which may help them maximize their quality of life (Prince et al., 2011).

The strongest risk factor for dementia is age, with prevalence in all over 65 s of between 6.4% and 7.1%, doubling with every 5‐year increase in age to 28.5% at 90 years and older (van der Flier & Scheltens, 2005). Additional, well‐evidenced non‐modifiable risk factors include sex (dementia is more common in women), and genetics (particularly APOE4; The PHG Foundation, 2019). Potentially modifiable risk factors include early childhood deprivation, lower level of education, midlife hypertension and obesity, late life smoking, depression, sedentary behavior and diabetes, which are thought to all give rise to a higher risk of dementia (The PHG Foundation, 2019). These potentially modifiable risk factors account for about 40% of dementias worldwide (Livingston et al., 2020).

### 1.2 How dementia is diagnosed at present

Diagnosis and medical care for people with dementia (PwD) typically starts with the recognition of dementia symptoms, often by PwD and their families, followed by a decision to seek help for the symptoms. The next step, in high‐income countries with extensive primary healthcare systems, is for the patient to be seen and symptoms recognized by primary care physicians (Wells & Smith, 2016). Cognitive assessment tools such as the Informant Questionnaire on Cognitive Decline in the Elderly are often used to assist healthcare professionals with assessing for potential cognitive decline (National Institute for Health and Care Excellence [NICE], 2018). Patients are then usually referred to specialist services for a full memory assessment and diagnosis. This may take place in neurology, psychiatry, geriatric or multi‐disciplinary assessment clinics, and involves batteries of cognitive tests and some form of brain imaging. There is no single operational definition of dementia, so diagnostic thresholds differ in different contexts (Aldus et al., 2020). Post‐diagnosis care may revert to the primary care provider or continue in a specialist setting, and may include medication to help symptoms of cognitive disfunction, social support, or care provision for activities of daily living.

### 1.3 The dementia diagnosis gap across the world

National dementia strategies such as those implemented in the United Kingdom and elsewhere (Alzheimer Europe, 2021; Alzheimer's Disease International, 2021; Banerjee, 2010) have prioritized increasing rates of dementia diagnosis. This is driven by the assumption that treatment, care and support for dementia‐related symptoms can only be offered after a formal diagnosis has been made. UK national dementia strategies, first published in 2009, have aimed to improve diagnostic rates, targeting 67% of PwD having a formal diagnosis recorded in their primary care electronic patient record. This was achieved by 2017, although the estimated rate of diagnosis has subsequently declined (NHS Digital, 2022; NHS England, 2017).

Dementia is thought to be under‐diagnosed globally, with one systematic review finding the pooled rate of undetected dementia was 61.7% (Lang et al., 2017), and rates of under‐detection higher in China and India compared to North America and Europe. In addition, when dementia is recognized, it is often at a late stage. For example, Aldus et al. (2020) found an average time of 3.5 years from reaching clinical criteria for diagnosis to getting a clinical diagnosis in a sample of PwD in the United Kingdom. They found that PwD living with a partner were twice as likely to be diagnosed than those living in other settings.

### 1.4 Why would we want to detect dementia earlier?

**FIGURE 1:**
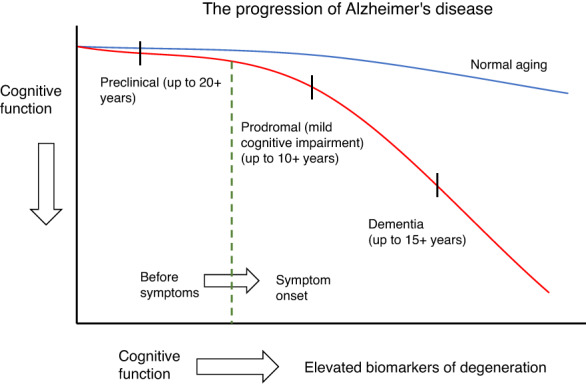
The continuum of Alzheimer's disease, where the preclinical phase may last for up to 20 years. *Source*: Adapted from Laver et al. (2016) and Gale et al. (2018).

Under‐detection and late detection have been drivers of efforts to improve the ability to detect dementia more effectively and efficiently. In addition, this is supported by a clinical model of disease that sets out a hypothetical linear and unidirectional model of progression, as shown in the case of Alzheimer's disease in Figure 1.

**FIGURE 2:**
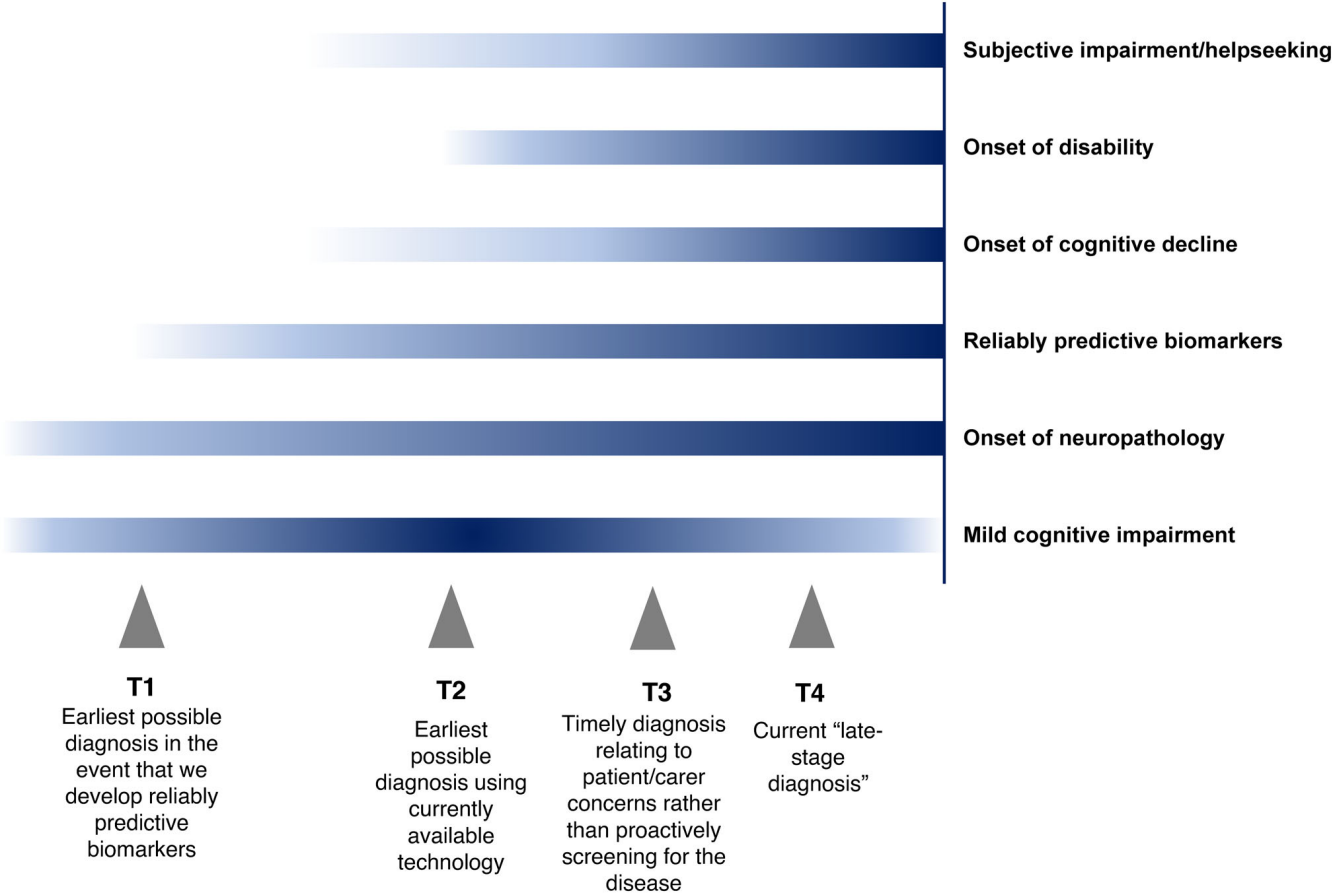
Diagram showing how opportunities for intervention intersect with possibilities for detecting dementia. *Source*: Adapted from Dubois et al. (2016).

The continuum model suggests that for any intervention to be successful in slowing or preventing dementia, it must be started much earlier than the detection methods currently implemented in clinical practice allow. It posits that the critical period for some kind of effective clinical intervention, and thus the ideal time for diagnosis, may lie between the earliest point at which the diagnosis *could be made*, and the time at which diagnosis *is currently made* (Figure 2). We summarize three reasons for investing in methods to diagnose dementia at earlier time points than is currently possible.

#### 1.4.1 Reason 1: Disease modifying treatment—Could we detect changes early enough to prevent, slow down, or reverse the neuropathology?

The long duration and heterogeneity of neurodegenerative process before symptoms show may explain why many drug interventions for dementia have failed to live up to expectations in real‐life contexts, as they have been tested on patients with a formal diagnosis of dementia, thus well‐established disease (Gauthier et al., 2016; Ritchie et al., 2015). However, should disease‐modifying or ‐reversing drugs become available for some dementias, they may have maximum efficacy when applied before the disease has clinically manifested and extensive and irremediable damage has occurred (Prince et al., 2011). Tools for detection aim to facilitate the identification of individuals who are in this potential zone for intervention and differentiate dementia from normal aging before the symptomatic phase.

Tools currently in routine use include structural brain scans (usually MRI, which is preferable to CT scans), which can identify neurodegeneration in the brains of PwD. However, particularly in the case of Alzheimer's disease, there is also increasing use of modalities such as positron emission tomography (PET) imaging, single photon emission computed tomography (SPECT) and cerebrospinal fluid (CSF) analysis. These aim to capture disease progression along the model trajectory of Alzheimer's disease shown in Figure 1, that involves the gradual accumulation of beta‐amyloid rich neuritic plaques and neurofibrillary tangles up to 20 years before symptom onset (Gale et al., 2018). PET imaging, CSF markers and future blood‐based biomarkers may also allow the identification of neurodegenerative changes in the brains of people who are currently asymptomatic or mildly symptomatic (Hampel et al., 2018). Given that many drugs in development target these biological markers, using them for early detection may be key to getting the best outcomes (Cavedo et al., 2014).

Importantly though, such tests are not definitively diagnostic, as neuritic plaques are also found in the brains of those who die without dementia, such that tests for amyloid or tau levels cannot give a definitive view of whether someone is living with the early stages of dementia or not, rather these tests corroborate a wider clinical picture (Neuropathology Group & Medical Research Council Cognitive Function and Aging Study, 2001). Moreover, despite the approval of Aduhelm by the US FDA, there is evidence that pharmacological effects on amyloid pathology have limited cognitive benefits, and indeed, there are suggestions that such effects are in fact absent (Ackley et al., 2021; Richard et al., 2021). Thus, at the present time it is not clear when, or even if, a successful, disease modifying agent for dementia would become available.

#### 1.4.2 Reason 2: Identify target populations for trials of dementia treatments

While the first motivation for early detection relates to the identification of those most likely to directly benefit, the second is that without identifying individuals at “early” stages of disease, it is impossible to accurately assess the value of new therapies that may either benefit an individual now or similar patients in the future. As outlined above, it has been hypothesized that new target compounds for modifying the disease trajectory of dementia will work best at the earlier stages of neurodegeneration (Gauthier et al., 2016; Jack et al., 2018). To evaluate the effectiveness of such compounds, it is necessary to identify patients reliably who have signs of neuropathology, which correspond with early‐stage disease, to take part in trials. For example, amyloid biomarkers are increasingly widely used to identify “at‐risk” participants for Alzheimer's clinical trials (Alber et al., 2017), with PET imaging used extensively to assess the eligibility of potential participants in the large Phase III Anti‐Amyloid in Asymptomatic Alzheimer's (A4) trial of Eli Lilly's solanezumab. “Trial‐ready” platforms such as the European Prevention of Alzheimer's Dementia (EPAD), the Trial Ready Cohort for Prevention of Alzheimer's Disease (TRC‐PAD) and the Japanese Trial‐Ready Cohort (J‐TRC) aim to facilitate the rapid inclusion of participants with known biomarker status (Aisen et al., 2022). However, these approaches are costly and inefficient when applied at a single trial level—in the case of the A4 study, 71% of people who underwent PET imaging did not meet the inclusion criteria (Meyers & Carrillo, 2020). There is thus a desire for alternative approaches to identify individuals most likely to meet biological inclusion criteria, including the use of blood‐based and digital biomarkers for disease detection (Gold et al., 2018).

#### 1.4.3 Reason 3: Help people live well with dementia—Timely access to information, advice, support and appropriate care

Even where no disease‐modifying pharmacological treatments exist, there may still be benefits to patients and families in early detection of disease. While the patients' symptoms are still mild, or even before symptoms emerge, they have the opportunity to clarify the details of their condition with their clinicians and caregivers, understand its prognosis and trajectory, state their wishes for care, and organize their finances for their future needs (Prince et al., 2011). There are also some late‐life modifiable risk factors for dementia, such as smoking, physical activity, social isolation and mental health (Prince et al., 2014; The PHG Foundation, 2019). It is possible that if changes associated with future risk of dementia are detected very early, modifying these risk factors could slow disease progression and/or help people with dementia to live longer with a higher quality of life, by, for example, minimizing comorbidities. Several self‐management interventions have been trialed with people with early‐stage dementia to help improve health behaviors and quality of life (Buettner & Fitzsimmons, 2009; Quinn et al., 2016). To date, however, the evidence for the effects of such programs remains limited. Among those without symptoms, studies of amyloid risk communication have found some effect on intended and actual changes in lifestyle and advance planning (Largent et al., 2020), although the evidence from the communication of genetic risk information suggests that such lifestyle changes may be unlikely without support (Hollands et al., 2016). Importantly, the only substantial clinical trial to date of dementia screening in primary care found no differences in healthcare utilization, advance care planning, or disease recognition by physicians after a year (Fowler et al., 2020).

## 2 CURRENT TECHNOLOGY FOR EARLY DETECTION OF DEMENTIA

In reviewing technology for early detection, it is important to separate two approaches. The first is risk prediction, that is, the identification of groups of people who are at high risk of developing dementia in the future, but do not currently have detectable or diagnosable disease as defined by current diagnostic criteria. This might include, for example, identifying people who have various lifestyle‐related risk factors or who carry the ApoE e4 risk allele that puts them at increased risk of disease. This should be distinguished from early detection, which is the focus of this chapter, in the sense of methods to identify individuals who have markers or signs associated with dementia‐causing diseases, earlier than currently happens in the clinic.

### 2.1 Blood‐based versus digital biomarkers

Given the limits of the “fluid” markers described above, there is growing interest in alternative approaches, including blood‐based markers, and digital tools, which form our focus.

“Digital” biomarkers are defined as objective, quantifiable physiological and behavioral data that are collected and measured by means of digital devices such as portables (e.g., tablets, smartphones), wearables (smart watches), implantables, digestibles or passive sensors embedded in homes or cars (Babrak et al., 2019). Digital biomarkers denote the use of a biosensor to collect objective data on a biological (e.g., blood glucose, serum sodium), anatomical (e.g., mole size), or physiological (e.g., heart rate, blood pressure, gait, level of physical activity) parameter followed by the use of algorithms to transform these data into interpretable outcome measures (Dorsey et al., 2017) .

### 2.2 Types of digital biomarkers

There are a range of different out‐of‐clinic technologies which could generate data to inform algorithms tuned for early detection of cognitive impairment and dementia. These include tools adapted from existing cognitive assessment measures and novel assessments that make use of the capacities of smartphones and similar tools through gamification, virtual and augmented reality and image‐based assessments. In addition, a range of more “passive” forms of data collection aim to make use of sensors in wearables, in‐home or in‐car devices, and data derived from use of non‐dedicated technological devices (e.g., capturing patterns of mouse movements during computer use; Piau et al., 2019).

#### 2.2.1 Screening tools on mobile/ICT devices

The first approach to developing digital biomarkers for the early detection of dementia is to put established or validated cognitive screening tests onto apps and websites so that patients can complete these tests on a regular basis (Thabtah, Peebles, et al., 2020), and potentially outside the clinic. Bringing these cognitive tests out of the clinic and into patients' everyday life allows the influence of daily life activities on cognition to be captured, an effect which is not picked up when tests are only administered in the artificial environment of the clinic (Allard et al., 2014). Between 2010 and 2015, 31 computerized instruments were described in the literature, these were presented to patients on PCs, laptops and tablets and using both touchscreen and keyboard inputs; however, validity and reliability of these tests were rather poorly reported (García‐Casal et al., 2017).

More recently, tests have been incorporated into mobile phone applications (apps). In their review of dementia screening apps, Thabtah et al. found 20 different apps which presented established cognitive screening tools such as the mini‐mental state examination (Thabtah, Peebles, et al., 2020). Almost all had been validated against clinical populations and showed high accuracy (sensitivity range: 38%–100% and specificity range: 40%–100%). Few apps employed machine learning to process the data generated, although some employed artificial intelligence (AI) to analyze drawings (e.g., of a clockface, although no further details were given about AI techniques). However, Thabtah et al. argue that such applications need to focus on the entire cognitive functioning capacity to meet DSM‐V diagnosis standards (Thabtah, Mampusti, et al., 2020). They suggest that such comprehensive assessment apps would save time in clinic and speed up formal diagnoses.

#### 2.2.2 Smartphone‐native applications

In addition to porting existing tests into digital formats, a range of digital biomarkers also make use of the novel capabilities afforded by smartphones and tablets. These aim to address concerns that the results of cognitive assessment tests may be confounded by age, educational level, and language ability (Cordell et al., 2013). Digital biomarkers may therefore offer the opportunity to use language‐agnostic assessments, as in the Integrated Cognitive Assessment (ICA; Modarres et al., 2021), currently undergoing validation in the UK National Health Service (NHS), or incorporate augmented reality assessments (Meier et al., 2021). Data produced by the ICA was able to detect cognitive impairment with accuracy (AUROC) of 0.81 using a logistic regression model (Modarres et al., 2021). Other researchers have proposed alternative approaches such as gamification (Tong & Chignell, 2014) and virtual reality (Banville et al., 2010; Nolin et al., 2013; Plancher et al., 2012) in which users play games which can assess domains of cognition without relying on language. Games can be used to assess a range of areas of cognition, with high accuracy and acceptability or usability, their discriminative accuracy has been assessed with support vector machine, linear regression and random forest techniques (Valladares‐Rodriguez et al., 2017; Valladares‐Rodriguez et al., 2019). Accuracy and acceptability factors must both be considered in a test presented as a game. Also of interest are smartphone apps which “passively” measure user activity, such as typing habits, step counts, or location tracking (Sabbagh et al., 2020). While a number of these apps have moved from research into commercialization and are undergoing validation, none have been widely introduced into health services, and there is limited evidence of their cost‐effectiveness (Shore et al., 2022).

### 2.3 Passively collected data

As well as approaches that rely on the active engagement of the person being assessed, digital biomarkers present the opportunity to draw on the wider streams of data generated by people in the course of their everyday lives and use of technology.

#### 2.3.1 Wearable and mobile sensors

The widespread adoption of smartphones and devices such as the Apple Watch and Fitbit has suggested the possibility of collecting data about an individual's real world cognitive function and providing repeated, real‐time measurements taken in real‐life settings, as opposed to cognitive assessments and brain scans administered in the clinic only once or at spaced intervals (Dagum, 2018). Major programs, including collaborations between Apple, Evidation and Eli Lilly (Chen et al., 2019), Apple and Biogen (Intuition Study), Alzheimer's Research UK's Early Detection of Neurodegenerative Diseases (EDoN; EDON Initiative, 2021; Frey et al., 2019), RADAR‐AD (Muurling et al., 2021) the MRC Deep and Frequent Phenotyping study (Koychev et al., 2019) and the SENDA trial (Müller et al., 2020) are exploring the potential of a range of digital technologies to collect data from community dwelling adults. Measures captured by digital devices may include sleep, neural activity, cognition, speech and language, gait, heart rate, fine motor skills and physical activity, with the aim of collecting a digital picture of individual's daily function, linking fluctuations in memory performance with specific daily life activities or experiences and identifying subtle changes suggestive of cognitive decline (Kourtis et al., 2019). To date, however, algorithms to process sensor or wearables data to make accurate classifications for cognitive impairment or dementia are still in their early stages, but some success has come from using, for example, Extreme Gradient Boosting algorithms, to enable handling of missing data and to produce explainable results, and achieving accuracy of nearly 90% (by AUROC; Chen et al., 2019).

#### 2.3.2 In‐home sensors

A further aspiration of digital assessments since the late 1990s has been the assessment of functional performance in real‐life environments to help identify and clarify difficulties in situ (Abowd et al., 2002). “Smart” environments have embedded sensors (e.g., infrared, magnetic or electric) or cameras, so that the task of capturing individuals' functional performance is not influenced by the act of observing them, and so that measurements can be taken regularly and “passively,” providing longitudinal data to identify change. Developers of such measures also point out that they are unobtrusive and non‐intrusive (Piau et al., 2019); once an individual's home is fitted with the sensors, they do not need to remember to put them on or take them out with them, as is the case with wearables or mobile devices.

To date, studies have found evidence of differences between people with cognitive decline and healthy controls on a range of such measures, including walking speed, level of activity (i.e., going out of the house), movement or awakenings during the night, frequency of daily ICT device usage, and regularity of medication intake (Lussier et al., 2019; Rawtaer et al., 2020). Again, the development of algorithms to differentiate people with intact cognition from those with cognitive decline, from in‐home sensor‐based data, are at early stages, but results are promising. Support vector machines and random forest algorithms have been trialed to discriminate between people with MCI and healthy controls, using at‐home sensor data, achieving AUROC of 0.97 (Akl et al., 2015).

#### 2.3.3 In‐car sensors

Around one third of drivers with dementia continue to drive in the early stages of the disease, while cognitive deficits are mild (Silverstein, 2008). Making the decision to stop driving can be difficult for individuals, signifying a non‐reversible loss of independence and mobility. Advances in sensor, computing and communication technologies mean that information about a person's driving performance can be collected and analyzed unobtrusively. There may be commonalities in driving performance changes in dementia patients, which could indicate the early onset of dementia. Eby et al. found several differences in driving behaviors in participants with early‐stage dementia and an age‐matched comparison sample, such as driving more slowly, driving shorter distances, and to fewer destinations (Eby et al., 2012). These differences could potentially be used, against an individual baseline, to detect the early stages of dementia (Bayat et al., 2021).

### 2.4 The use of AI to accurately interpret data in the clinic

The previous sections have explored out‐of‐clinic technologies which could provide extra data about patient trajectories of cognitive and physical functioning to aid dementia detection. A further approach is to apply novel analysis methods to data which are already being collected in the clinic (e.g., Electronic Health Records [EHR] data) or in research cohorts such as the Alzheimer's Disease Neuroimaging Initiative (ADNI, 2017), with the aim of finding signals that can differentiate dementia patients from controls, and which may not be obvious to clinicians interpreting the data.

Clinical data can take various forms. The key data sources which have been explored for dementia detection are neuro‐imaging data (MRI, PET, SPECT and diffusion‐tensor imaging [DTI]; Teipel et al., 2015); EHR data covering medical diagnoses and history, comorbidities, sociodemographic and administrative variables; cognitive screening tests scores and neuropsychological test battery scores; genetic data; and transcribed speech. Many different forms of machine learning have been applied to these different data sources, and techniques can be largely grouped into supervised learning (classification into labeled groups), unsupervised learning (clustering) and deep learning (using multiple layers of nonlinear information processing to recognize feature qualities, generally classifying labeled data, but without revealing the detailed internal processes; Dashwood et al., 2021).

There have been several reviews of machine learning approaches to detect dementia in brain imaging (Ahmed et al., 2019; Pellegrini et al., 2018). Studies using the ADNI dataset, which includes fMRI brain imaging, dominate work developing prediction models for Alzheimer's disease (83% studies in one review on imaging; (Pellegrini et al., 2018) and 43.5% in another review drawing on wider sources of data (Goerdten et al., 2019)), the second most commonly used source is the Open Access Series of Imaging Studies (OASIS; Marcus et al., 2007). Historically, supervised learning using support vector machines (SVM) was the most commonly used approach to classify images into dementia vs controls (59% of studies in Pellegrini et al., 2018). Good performance was achieved with SVM, but predicting brain phenotypes and dementia subclassifications remained challenging (Ahmed et al., 2019). Clustering techniques such as principal component analysis offer some advantages such as low noise sensitivity and increased efficiency, and have shown promise in separating images in terms of brain dysfunction (Ahmed et al., 2019). However, the most accurate classification of brain images has been achieved through the application of “deep learning” to the task of discriminating between dementia patients and controls. Techniques have included feed‐forward neural networks (Akhila et al., 2017), including autoencoders such as randomized denoising autoencoder markers (Ithapu et al., 2015), and stacked auto‐encoders (Dolph et al., 2017; Ju et al., 2019); deep belief networks (Faturrahman et al., 2017); and convolutional neural networks (Murugan et al., 2021; Sarraf & Tofighi, 2016) including deep ensemble sparse regression networks (Islam & Zhang, 2017), and residual networks (Odusami et al., 2021). In general, deep learning models have achieved highly accurate discrimination between patients with normal cognition, mild cognitive impairment and dementia (ranging 80%–99% accuracy; Pellegrini et al., 2018). Studies have also developed models to predict who is at risk for future accumulation of tau proteins, the accumulation of which is associated with neurodegeneration and cognitive impairment (Giorgio et al., 2022). One of the drawbacks to the use of deep learning is the need for very large sets of labeled data. These models are also limited to detection of dementia in patients who are seen in hospital or specialist settings (and can afford the cost of a brain scan). Furthermore, the reliance on a limited number of datasets (mainly ADNI) presents substantial challenges for the generalizability of current prediction models.

Healthcare systems which generate electronic, longitudinal, routinely collected healthcare service use data on individuals (such as UK primary care) provide the basis for modeling trajectories of cognitive decline over time. Many future risk prediction models have been developed using electronic health records (EHR) data (Barnes et al., 2020; Ben Miled et al., 2020; Fu et al., 2022; McCoy et al., 2019; Nori et al., 2020; Reinke et al., 2022); fewer studies have used EHR data to detect existent dementia cases which were previously undiagnosed. To date, detection models have trialed simple classifiers such as logistic regression, random forest, and SVM (Ford et al., 2019; Jammeh et al., 2018; Shao et al., 2019), with accuracy (measured by AUROC) ranging from 0.74–0.91. Notably the lower accuracy presented by Ford et al. (AUROC 0.74) was achieved after the team removed features which would indicate that the GP had already recognized a memory problem, and started the underpinning diagnostic process for dementia, for example, recording a memory problem or making a referral to a memory assessment service. When these memory‐related features were subsequently included, and notably when free text clinical notes were also included, the model accuracy increased substantially (AUROC 0.84 with memory variables [Ford et al., 2020] and 0.94 with keywords from clinic notes [Ford, Sheppard, et al., 2021]). A review of using deep learning on EHR data (e.g., feed‐forward neural networks [FFNN], convolutional neural networks [CNN], and recurrent neural networks [RNN]), found wide paper‐to‐paper variability, in techniques and model accuracy, but suggested RNN might be the best approach on EHR data to model the sequences of events in longitudinal records (Ayala Solares et al., 2020).

Discrimination between dementia patients and controls has been successfully modeled using scores from cognitive tests and neuropsychiatric tests administered in the clinic. So et al. (2017) reported a two stage screening process using MMSE data to discriminate between normal cognition and cognitive decline, and a second stage using and CERAD‐K data (the Korean version of the Consortium to Establish a Registry for Alzheimer's Disease) to classify cognitive decline patients into Alzheimer's or MCI diagnosis. They used a range of methods for example, SVM, Naïve Bayes, Random Forest, Bagging, Bayesian Networks and Multi‐layer Perceptron (MLP; a type of feed‐forward neural network). In the screening phase using MMSE data, MLP performed highly accurately (F1 measure 0.97), and in the second diagnostic phase, SVM had highest accuracy (F1 measure 0.74). In Chinese data, Zhu et al. (2020) used the Cognitive Abilities Screening Instrument (CASI) and Montreal Cognitive Assessment (MOCA), in random forest, Ada‐boost, Logit Boost, neural network, naïve Bayes and SVM models, and found naïve Bayes offered the best discrimination with F1 measure of 0.87.

Some clinical tasks, such as describing pictures, generate patient speech data which can be transcribed. Features can be extracted using natural language processing (NLP) to generate structured data, which can then be fed into ML classifiers. In one study using the Pitt corpus (Becker et al., 1994), using logistic regression models, discrimination between dementia cases and controls was achieved with about 88% accuracy (Roshanzamir et al., 2021). Another study using the ADReSS Challenge dataset (Luz et al., 2021) compared conventional classifiers with a deep learning model incorporating BERT (Bidirectional Encoder Representations from Transformers; a pre‐trained, open source deep learning framework for NLP [Devlin et al., 2018]), and found that the BERT deep learning models outperformed the conventional ML models, but by only a small margin (BERT F1 measure 0.81, with SVM performing at 0.79; Balagopalan et al., 2021).

Finally, some teams have trialed modeling multi‐modal clinic data from different sources, such as MRI, genetic and neurological examination data using deep learning (e.g., James et al., 2021; Qiu et al., 2022; Venugopalan et al., 2021). Some approaches have used staged models, to separate out cognitively impaired individuals first and then derive a refined diagnosis of AD versus MCI on the selected patients in a second stage (Qiu et al., 2022; Zhou et al., 2019). Some studies have shown that integrating multi‐modal data, for example genetic data, clinical test data, plus imaging, as well as using deep learning rather than “shallow” models, produces better performance than single modality data (Venugopalan et al., 2021).

## 3 WHAT ARE THE POTENTIAL ETHICAL ISSUES?

Many of the early detection methods using digital biomarkers that are described above incorporate machine learning or AI methods. Their implementation in an ethical and responsible manner thus presents challenges related to the source of data and method of analysis, and the field of application.

Guidance for reflection on “AI ethics” in the clinical environment has been set out by a number of bodies, most notably the World Health Organisation and the European Commission (European Commission High‐Level Expert Group on AI, 2019; World Health Organisation, 2021). The approaches commonly rest on broadly agreed principles that include core principles of medical ethics—benevolence, nonmaleficence, justice, respect for autonomy—and a specific emphasis on transparency, explainability, interpretability, intelligibility, responsibility and accountability (Solomonides et al., 2022). As these technologies are integrated in health care, models of AI governance thus emphasize the need for fairness, trustworthiness, transparency and accountability (Reddy et al., 2020). Such considerations are particularly important in light of the unprecedented amount of information about users that is captured by digital technologies, the potential invasion of privacy that results, and potential harms from data breaches (Perakslis & Ginsburg, 2021).

Guidance on the ethics and governance of AI tools has practical relevance for their successful implementation. For example, there is some evidence that clinicians are more likely to trust AI which is explainable, that is, it is clear to the clinician which input features were weighted in the AI model to justify the output (Reddy et al., 2020). However, new deep learning models (usually neural networks) are currently much less explainable than other forms of machine learning, yet to date, give the highest accuracy of dementia early detection models, creating a tension for clinical implementation. The integration of AI tools into the clinical workflow also raises questions about accountability and fairness (Reddy et al., 2020), such as in attributing liability for errors resulting from the use of AI devices in the clinical context or conversely, from their non‐use. Robust guidance is needed over the circumstances in which a clinician would or should feel confident to dismiss the AI result, or how they can best explain a “blackbox AI” output to a patient.

However, in addition to wider questions around AI in healthcare, the implementation of automated methods for the early detection of dementia requires engagement with domain‐specific concerns. These include both the *disease context* and the nature of the *clinical practice or service* into which tools will be introduced. In this case, these are the prediction of dementia and the implementation of screening programs.

Debates about the ethics of dementia risk prediction have been current since at least the identification of the ApoE e4 allele associated with increased susceptibility to Alzheimer's disease in the early 1990s (Corder et al., 1993; Post, 1996). These debates have primarily focused on the value of knowing or not knowing test results and the impact of this knowledge on an individual's view of their future. These discussions have evolved through deliberations around the use of tools such as PET amyloid imaging or cerebrospinal fluid biomarkers and remain vital as digital biomarkers emerge (Harms et al., 2022; Karlawish, 2011; Molinuevo et al., 2016; Piau et al., 2019).

Digital biomarkers, however, present slightly different challenges, not least because of the possibility they offer of detecting signs of neurodegenerative disease before the onset of the symptoms at scale, using widely available technology and in a way that may not be directly controlled or initiated by a clinician. Instead, either a patient‐user or the developer of the biomarker technology may have control over its use. These factors point to the need for a robust framework for contemplating both the practical and ethical questions associated with digital tools for early detection.

One robust approach to considering these interlinked questions of ethics and practice is provided by the lens of population screening. In their seminal 1968 report for the World Health Organisation (Wilson & Jungner, 1968), Wilson and Jungner described the object of screening for disease as *to discover those among the apparently well who are in fact suffering from disease*, a conceptualization which fits well the paradigm of early detection. They set out 10 principles in the form of normative statements, which have become paradigmatic in evaluating whether a screening program is desirable, and whether it is likely to achieve the ethical goal of producing more benefit than harm:

1. The condition sought should be an important health problem.
2. There should be an accepted treatment for patients with recognized disease.
3. Facilities for diagnosis and treatment should be available.
4. There should be a recognizable latent or early symptomatic stage.
5. There should be a suitable test or examination.
6. The test should be acceptable to the population.
7. The natural history of the condition, including development from latent to declared disease, should be adequately understood.
8. There should be an agreed policy on whom to treat as patients.
9. The cost of case‐finding (including diagnosis and treatment of patients diagnosed) should be economically balanced in relation to possible expenditure on medical care as a whole.
10. Case‐finding should be a continuing process and not a “once and for all” project.

While these principles often form the basis for governmental or quasi‐governmental programs for the assessment and governance of screening programs (Sturdy et al., 2020), they have wider relevance to any systematic attempt to identify those with hitherto unidentified disease. As such, they are highly relevant to early detection programs—whether established by health systems, health insurers or in other settings, for example by technology developers. To date, this wider relevance has rarely been recognized in discussions of digital health, including in the case of dementia (Gunnarson et al., 2021). Below, we consider how these principles apply for the paradigm of dementia early detection using digital biomarkers and automated detection methods, and emphasize the ethical issues brought to light by each principle.

### 3.1 The condition sought should be an important health problem

It is clear from the increasing numbers of people with dementia globally, the aging population across the world, and the cost or burden of high‐quality care for people living with dementia, that dementia constitutes an important health problem. Consequently, in the case of symptomatic disease, this condition is largely met. However, as discussed below, this is more complex in the case of detection prior to the emergence of symptoms or in mildly symptomatic disease, which, if widely accepted as health problems, may cover a substantial population worldwide (Brookmeyer et al., 2018; Gustavsson et al., 2017). In order to establish this, and as elaborated below, it is critical that criteria (4) and (7) related to the understanding and identification of the disease trajectory, from early or latent stage through to full dementia, are met.

### 3.2 There should be an accepted treatment for patients with recognized disease

Screening programs can only be justified if there is an accepted treatment for patients with recognized disease. Unless an acceptable treatment can *currently* be provided which improves the prognosis of the disease, and which gives better outcomes due to the condition being found at an earlier stage, there can be no advantage to the patient of intensive case‐detection in an otherwise well population (Wilson & Jungner, 1968).

As discussed above, no treatments that effectively modify disease trajectory for dementia are available (Ackley et al., 2021; Richard et al., 2021). Consequently, large scale detection programs do not yet meet this criterion. As a result, while there may be cases in which individuals opt in to something approximating early detection, notably when choosing to take part in a clinical trial (Kim et al., 2015), it would be unethical to proceed with systematic or population‐based early detection of dementia on the basis only that a disease‐modifying treatment may become available in the future.

This is also an important consideration for risk prediction: before widespread programs involving individual risk prediction models are rolled out beyond research settings it is essential that evidenced risk reduction strategies at an appropriate life stage are available (Weatherby & Agius, 2018). As the World Health Organisation guidelines on risk reduction recognize, evidence in most areas of risk reduction for dementia is currently moderate or low, particularly for older and mildly symptomatic populations (World Health Organisation, 2019).

### 3.3 Facilities for diagnosis and treatment should be available to all

Screening programs identify a range of people who are likely to have the disease, for whom additional follow up tests are needed to establish a diagnosis. Resources must be allocated in the health service at the time of the implementation of an early detection program to manage the flow of patients referred for diagnostic assessment. However, mental health services in many health systems are drastically underfunded (Bannister, 2021) and cannot cope with current demand (World Health Organisation, 2017). While the hope is that early detection will reduce healthcare costs in the long term (see 9 below), current systems are unable to absorb a wave of patients channeled through to memory assessment services following screening. Consequently, for early detection programs to be effective and ethical there should first be a sufficient commitment of resources to dementia diagnostic and support services (Brayne & Kelly, 2019). This is an important factor in the development of digital biomarkers which may be inexpensive to administer but impose costs throughout the health system.

Additional consideration should go toward the psychological support needed when dealing with information about personal risk of dementia. At the current time, dementia is a progressive disease with no disease‐modifying treatment options and with implications for future health and mental capacity. To date, evidence related to the impact of receiving predictive information related to dementia while asymptomatic (e.g., based on genetic or biomarker tests) has been accompanied by counseling and professional support before and after testing (Goldman et al., 2011; Harkins et al., 2015). While such support may not necessarily be required long‐term, there is limited evidence of the benefit versus harm consequences of unsupported return of results (Frederiksen et al., 2021). Further, upon receiving results, people may also require and (reasonably) expect some level of support, monitoring and follow‐up (Milne et al., 2018); a service that again should be factored into cost predictions for the systemic impact of digital biomarkers (Schmutte et al., 2022).

### 3.4 (4) There should be a recognizable latent or early symptomatic stage and (7) the natural history of the condition, including development from latent to declared disease, should be adequately understood

Contemporary models of Alzheimer's disease posit a linear model of disease characterized by early and prodromal phases of disease that can be identified by changes in biomarkers or subtle alterations in cognition, mild cognitive impairment, and/or psychological and behavioral symptoms. While this linear model holds at a population level, the future trajectory of any individual patient's cognition from mild impairment to dementia cannot yet be accurately predicted (Hu et al., 2017). Patients in the early stages of disease may be labeled as having mild cognitive impairment (MCI), subjective memory complaints (SMC) or subjective cognitive decline (SCD). Each of these confers an elevated risk of developing dementia in the future (Slot et al., 2019). However, many patients in these categories will stay stable or show improvements, with only around 10% of those labeled as having MCI being diagnosed with dementia in each following year (Bruscoli & Lovestone, 2004), and up to 18% reverting to normal cognition (Canevelli et al., 2016). Machine learning and AI analysis techniques could likely contribute to understanding which patients with MCI are at highest risk of conversion to dementia; however, tests to gather the data needed for current predictive methods remain invasive and expensive (Eckerström et al., 2013; Gelosa & Brooks, 2012; Gomar, 2011; Zhang & Shen, 2012).

In sum, while there is a prodromal or latent phase in dementia, this needs to be better understood before the introduction of screening tests. It is not easy to determine the future course of disease for an individual with mild cognitive impairment. What makes one person move from MCI to dementia and another revert to normal cognition is not well understood.

### 3.5 (5) There should be a suitable test or examination

The principle of there being a “suitable” test is arguably the hardest to meet in the paradigm of early detection of dementia. A “suitable test” should be (a) highly accurate, (b) minimally invasive and (c) low cost.

Digital devices are clearly “suitable tests” in that they are minimally invasive and potentially low cost. However, although Wilson and Jungner suggest that “simplicity and ready application” (Wilson & Jungner, 1968, p. 33) may be enough to make a test suitable, in the case of dementia the potential consequences of screening for individuals and populations emphasize persistent and significant challenges related to accuracy.

As outlined above, where prevalence is low, the accuracy of a screening test, usually given as sensitivity and specificity, must be extremely high not to produce more false positives than true positives. For example, a dementia detection method rolled out on all over 65‐year olds in the United Kingdom, where dementia prevalence is 7.1% (NHS England, 2017), with 95% sensitivity and 90% specificity (representative of some of the best current digital biomarker methods), would have a positive predictive value (i.e., the proportion of positive results which are true positives) of 42%. Thus 58% of individuals given a positive result would subsequently be found not to have dementia.

As noted, while digital biomarkers offer some promise, their accuracy is not yet well established. Few digital health technologies have been rigorously shown to be equivalent to standard, accepted measurement devices (Perakslis & Ginsburg, 2021). The more accurate AI approaches rely on invasive brain imaging data and/or blood/CS fluid biomarkers and very few of the passive digital biomarker methods can yet offer sensitivities and specificities over 90%. As such, these tests do not *currently* meet the requirements of being highly accurate. In fact, the widespread and indiscriminate implementation of these methods to automatically detect dementia from multiple passively generated sources of data without knowledge of prior health status may lead to an inflation of the false positive risk (Ienca et al., 2018). In implementing digital screening tools therefore, those responsible should consider the potential rate of false positive results and the consequent risk of harm to these individuals, and plan how to mitigate it.

The possibility that implementation of AI will widen health inequalities must also be addressed and monitored (Walsh et al., 2020). There is a risk that algorithms for early detection will work less accurately when making predictions on some groups than others, and that this may not be evident to those using them. Such algorithmic bias is inherited from the social patterns reflected in training data, including how data is created or collected, and is increasingly well evidenced in medical applications beyond dementia (Obermeyer et al., 2019). Such bias refers to any application of algorithms that compounds existing inequities in socioeconomic status, race, ethnic background, religion, gender, disability, or sexual orientation.

Many of these existing inequalities are evident in the case of dementia and dementia research. Hence, for example, in the United States, African‐Americans are at greater risk of dementia than whites and Asian‐Americans (Mayeda et al., 2016), as are those living in areas of the United Kingdom with higher levels of deprivation (Matthews et al., 2016), and immigrants to the Netherlands with non‐Western ethnic backgrounds (Parlevliet et al., 2016). All have substantially higher risks of developing dementia, but may be under‐captured in both clinical and research data, due to systemic inequities in healthcare. People from minority ethnic groups in western countries are also under‐represented in widely used datasets such as ADNI, in part because of differences in how people engage with clinical services and research. Research datasets, sampled from different populations, are also heterogeneous and present challenges for validation and replication (Birkenbihl, Salimi, Fröhlich, Japanese Alzheimer's Disease Neuroimaging Initiative, & Alzheimer's Disease Neuroimaging Initiative, 2022). Unsurprisingly, many dementia risk models—even developed without automated approaches—already do not translate across cultural settings (Stephan et al., 2020). These limitations and inequalities may be compounded in the case of automated systems.

### 3.6 (6) The test should be acceptable to the population

As suggested above, the acceptability of a dementia screening test to the target population is by no means a given. People have mixed views on whether they would want to know if they currently have dementia (Corner & Bond, 2004; Marzanski, 2000) or are at increased risk of developing it in the future, with some research suggesting that only patients with severe dementia uniformly want to know their diagnosis (Jha et al., 2001).

Overall, it has been estimated that between half and 90% of people may be interested in knowing the results of a “predictive” or “reliable” test of whether they are likely to develop dementia, with numbers varying across countries (Caselli et al., 2014; Gooblar et al., 2015; Ott et al., 2016; Wikler et al., 2013). In the United Kingdom, two waves of Alzheimer Research UK's Dementia Attitudes Monitor have suggested that about three‐quarters of people would like to be told their personal risk of getting dementia in the future—and a similar proportion would be willing to use smartphone apps or wearable devices that might help them understand their dementia risk (Alzheimer Research UK Dementia Statistics Hub, 2021). However, given the current limitations of knowledge described above, information provided at this time would be highly uncertain—a factor that decreases interest in learning personal risk (Gooblar et al., 2015; Milne et al., 2018).

Dementia remains highly stigmatized—half of UK adults say that it is the condition they fear getting most (Alzheimer Research UK Dementia Statistics Hub, 2021). In the case of older adults without overt symptoms, some express fear and worry about dementia, feeling that “there was little that could be done” (Corner & Bond, 2004), and as a result were unlikely to seek health care advice or a diagnosis for memory problems. For some, this may result in fatalism—the view that dementia is an inevitable part of aging, or that nothing can be done to reduce risk, views which evidence suggests may be particularly common among lower socioeconomic groups and racial and ethnic minority groups (Alzheimer Research UK Dementia Statistics Hub, 2021). In this context, a screening program would pick up more people who would not otherwise seek a diagnosis—and for whom the perceived value of a diagnosis may be less (Brayne & Kelly, 2019).

There are few concrete examples of screening programs in practice. In a Iarge screening trial conducted in Indiana, 38% of those eligible to participate refused to do so, while two‐thirds of people who did participate and had positive results on screening for cognitive impairment refused subsequent diagnostic evaluation (Fowler et al., 2020). Patients refusing further testing were more likely to live alone and have a perceived stigma of dementia, while those who refused to participate raised concerns about potential emotional distress and fear of losing independence. This echoes concerns about losing health insurance cover, driving privileges, or employment and worry about the effect on family finances and emotions described in earlier work (Couteur et al., 2013). Others have raised concern about privacy and disclosure issues of personal data about dementia (Ienca et al., 2018). In practice, therefore, population screening for dementia may not be acceptable to the general public (Martin et al., 2015).

Moreover, the test or program of early detection should also be acceptable to clinicians who might have responsibility for administering or interpreting the test and discussing the result (see also below (8)). For clinicians, early detection tools that allow them to effectively identify those with memory complaints or mild cognitive impairment who are most likely to develop dementia may have value (Saunders et al., 2022). Clinicians may also perceive benefits associated with possibilities for life planning of healthcare, finances and care preferences (Schweda et al., 2018). However, they are also aware of risks associated with stigmatization, and discrimination in insurance, employment or by family members, as well as concerns about psychological distress in the absence of therapy (Schweda et al., 2018). In the United Kingdom, General Practitioners (GPs, also known as Primary Care or Family Physicians) are responsible for recognizing memory problems, referring the patient for further memory assessment and supporting the patient's holistic health care needs following diagnosis. It is most likely that any early detection method using digital biomarkers would be rolled out in primary care, in the community, or by private companies. However, GPs report resistance to making a dementia diagnosis early in the disease course (Iliffe et al., 2003) and adopt new predictive technologies in the clinic only when they meet certain criteria (Ford, Edelman, et al., 2021). GPs are more likely to use artificial intelligence as clinical decision support (CDS) when they are offered training in how to use it, the CDS is developed from a transparent evidence base, GPs have control over when to deploy it, and there is a clear communication guide and clinical pathway with which to follow up the result (Ford, Edelman, et al., 2021). GPs are also concerned about how communicating a bleak diagnosis may affect their relationship with their patient (Dhedhi et al., 2014). However, where GPs could have a well‐resourced diagnostic service and a dementia support package to offer screen‐positive patients, they would be, in the majority, favorable about systematic early dementia detection (Thyrian et al., 2016; Thyrian et al., 2018). A large majority of clinicians (86%) would also want clinical guidelines to accompany the testing program, with explicit information about risks and benefits of testing (Schweda et al., 2018). As a result, and as similarly concluded by Martin et al. (2015), clinician attitudes are likely to match those of the general public. Indeed, 69% of clinician‐respondents felt a screening program would only be useful or acceptable if there were effective treatment options (Schweda et al., 2018).

### 3.7 (8) There should be an agreed policy on whom to treat as patients

Who is considered as a patient in the context of dementia care has undergone a shift over several decades, reflected in evolving diagnostic criteria. Thus, while those with symptomatic dementia continue to be seen as the patients of memory services, since the early 2000s they have increasingly been joined by those understood to have mild cognitive impairment. More recently, the adoption of criteria delineating “preclinical” disease stages has led to the expanded global estimates discussed under (1) above.

However, both MCI and the most recent diagnostic classifications have proved to be contentious in both scientific and clinical practice, as well as being unclear for patients themselves (Gomersall et al., 2015; Saunders et al., 2022; Swallow, 2020). There is consequently uncertainty about whether and how the results of early detection should be communicated to patients, who should do this, and how patients should be supported to make decisions about what to do as a result. Thus, the clinical pathway associated with screening is not at all clear at the current time.

### 3.8 (9) The cost of case‐finding (including diagnosis and treatment of patients diagnosed) should be economically balanced in relation to possible expenditure on medical care as a whole

The balance of costs to the whole health and care system is critical question for the early detection of diseases leading to dementia, and for the development of technologies that aim toward this goal. As Wilson and Jungner pointed out in their elaboration of screening criteria, “*It is often considered that the detection of disease by screening will be economical of a country's resources*” (p. 35). Arguments in favor of early detection thus often pivot around the substantial economic cost of dementia care, particularly in later stages of disease. In the United Kingdom, the Alzheimer's Society, using figures from the MODEM study, estimates the cost of dementia care at £34.7 billion and projects an increase to £94.1 billion in 2040 (Wittenberg et al., 2019). In 2015, the global cost of dementia was estimated to be US$818 billion (Wimo et al., 2017).

It is important to note that the bulk of these costs are associated with direct or informal care. While some of these may be amenable to technological intervention (Knapp et al., 2016), they may not be *directly* affected by early detection tools. However, the ability to delay and slow the progression of dementia and control comorbid conditions may be facilitated by early detection, and this may have potentially significant economic impacts (Mattap et al., 2022). The US Alzheimer's Association, for example, has estimated that an intervention that delays the onset of dementia by 5 years may save $220 billion over that time (Alzheimer's Association, 2015). A more recent estimate, based on a projection of a 30% relative reduction of baseline progression rates from MCI to mild dementia and from mild to moderate dementia, suggests a $2.62 trillion benefit over 20 years in the United States, much made up of the gain in quality‐adjusted life‐years (Prados et al., 2022).

However, in the case of specific tools for screening and case‐finding, the health economic arguments are currently limited by other factors discussed above, crucially the availability (and timing) of treatments, evidence about longitudinal disease trajectories and patient acceptability (Barnett et al., 2014; Gustavsson et al., 2017). As such, it is not clear how strong the economic arguments are in favor of early detection.

In addition to the wider economic impact of early detection, digital tools for early detection present additional questions related to consumer expenditure on early detection and its financial consequences for both those individuals and health systems. Tools for risk assessment and early detection are already being incorporated into health insurance programs and offered as apps to older adults—for example, in the EASIIT collaboration between pharmaceutical company Eisai and digital testing firm CogState (Cogstate, 2020).

The adoption of apps by consumers shows an emerging direct‐to‐consumer screening market. It highlights both the potential for lifestyle prevention but also the exploitation of older adults' concerns about dementia—in the case of dementia, concerns that were evidenced in the substantial penalties handed out by the US Federal Trade Commission to the company Lumosity for preying “on consumers' fears about age‐related cognitive decline, suggesting their games could stave off memory loss, dementia, and even Alzheimer's disease” (US Federal Trade Commission, 2016).

The proliferation of digital screening tests also has potential implications for healthcare systems. In the United Kingdom, these concerns have resulted in a joint position statement from the UK Royal College of General Practitioners and the British Medical Association on screening by organizations which have not been approved by the UK National Screening Committee (Royal College of General Practitioners, 2019). This statement highlights the growing private sector offer of screening tests unsupported by high quality trials, with 91% of doctors reporting having seen patients in NHS time to discuss private health screening results; with only 13% feeling that this was an appropriate use of NHS resources. It concluded that such discussions “can be an inappropriate use of NHS resources and can have a potentially significant negative impact on primary care.” As such, it recommended that GPs consider informing patients why such tests are not offered through the NHS, that the government and NHS ensure that health care providers are not put under obligation to follow up the results of these tests, and that regulators remind private providers of their responsibility not to put the NHS at risk of subsidizing private care and ensure private clinics arrange follow‐up at their own cost.

### 3.9 (10) Case‐finding should be a continuing process and not a “once and for all” project

The final consideration for those developing digital and automated tools for early detection relates to the longevity of the projects they facilitate. For screening to be of maximum value, it must continue to pick up the people who will benefit most from it through regular offers of examination. This is one argument in favor of the development of ongoing surveillance and health checks based in primary care or community settings.

However, for digital devices this criterion also raises questions associated with access, sustainability and obsolescence. For a digital tool to be able to deliver large‐scale, repeated assessments, it must be accessible to the widest number of people using available technology, supported by future technology and/or compatible with future tools. This is often little acknowledged, but was evident in the roll‐out of digital contact tracing devices for Covid‐19; the largest implementation of digital health solutions to date, but one which highlighted the existence of a digital divide both in terms of device ownership and internet access and in terms of the currency of these devices (Ada Lovelace Institute & The Health Foundation, 2021). This divide risks limiting the accessibility of technology for those who are most in need. There is commonly a tension for developers of digital health technology, who in some cases must choose between taking time to run trials to gain evidence for effectiveness or non‐inferiority but risking obsolescence, or getting the product to market while it is still current and compatible with the devices currently in use but without much of an evidence‐base.

## 4 CONCLUSION

In this paper we have discussed the potential reasons for aiming to detect dementia earlier than is currently achieved, the emerging digital technologies which could achieve this, and the issues which arise when considering applying the early detection paradigm to a wide population. We have applied the lens of ethical screening programs to dementia early detection using digital biomarkers, and have found that efforts toward early detection of dementia fall short on a number of points.

The first issue is whether there is or can be any tangible benefit to patients or the population in identifying dementia in a pre‐ or early‐symptomatic population. This is currently doubtful, given the lack of treatments which change the course or outcome of the disease. No potential reasons for earlier detection are currently supported by a strong evidence base. Screening principles require there to be an available treatment which would change the course of the illness if the illness is detected at an early stage compared to a late stage. There is currently no good evidence that any dementia intervention changes the disease trajectory or prognosis. Many other screening programs show little to no benefit over a population, due to the harms associated with false positive results, and sometimes little benefit to outcomes of detecting asymptomatic disease (UK Government, 2021; World Health Organisation, 2020). Given the low evidence base for benefits for individuals of earlier detection of dementia, there is unlikely to be benefit across a wider population.

There is a prodromal phase for dementia during which patients experience some cognitive deficits but do not meet criteria for dementia diagnosis. However, it is neither clear nor certain that patients with mild cognitive impairment will progress to dementia, and some patients will regain normal cognitive function. While invasive tests of fluid biomarkers and brain scans can identify neuropathology prior to onset of symptoms there is no direct correspondence between detected neuropathology and level of cognitive impairment. Until we can accurately predict which patients will go on to develop symptomatic dementia from mild cognitive impairment or from detected neuropathology, it is difficult to say that there is a clear latent phase of dementia in which to focus disease‐modifying treatment efforts.

A potential reason to detect disease earlier is so that people can be aware of their condition and receive a prognosis at a time when they are able to plan and express their wishes for the future. This may be beneficial for family caregivers and patients, but this benefit would likely only accrue for people who were keen to know their dementia status. By extending early detection to a wider group of people, there is the risk of including people who do not wish to know their disease status or prognosis (Brayne & Kelly, 2019). If these technologies are state or health insurance funded and rolled out to a population, many individuals may be unwilling to engage or may not want to know their dementia status. If technologies are direct‐to‐consumer products offered by private companies, there is a potential risk of entrenching inequalities by means of cementing the digital divide between people who embrace both new technologies and health knowledge and awareness, and those who do not.

We reviewed the current and emerging technologies utilizing digital biomarkers or applying machine learning techniques to clinical data to detect patients with dementia at earlier time points. Screening criteria require that there should be a suitable and acceptable test for the condition being screened.

We characterized “suitable” as being low cost, non‐invasive and highly accurate; three conditions which are currently hard to meet in the current digital biomarker domain. While applying deep learning to extensive batteries of clinic data has started to produce highly accurate discrimination between dementia and cognitively intact patients, the kind of noisy data generated from less‐invasive and less costly passive sensors or wearables does not allow such accurate classification. The risk of generating many false positives with associated real risk of harm, must be seriously considered in this paradigm, given the current low accuracy of non‐invasive means of data collection. A key challenge relates to the potential for algorithmic bias. Reducing this will require datasets that are more representative of the target clinical population, and attentive to the social, cultural and economic factors that shape clinical care and thereby generate the data on which AI is trained or validated. This requires commitment of suitable resources, and a recognition that socially and ethnically diverse research teams may be necessary to effectively identify flaws and potential bias in the design and functionality of automated systems.

It is by no means a given that digitally enabled screening of the population for dementia would be an acceptable activity; not to patients, and neither to clinicians. One population who may benefit from improved characterization of prognosis are those patients with an MCI diagnosis. Knowing their future dementia status might be of real benefit to this cohort, and the chance of accurate prediction, while minimizing false positive risk, is more likely in a focused patient group at high risk.

Even without an approved disease‐modifying treatment, PwD have a number of care needs greater than equivalent age patients without dementia. Psychological screening and support would need to be available to help patients navigate what is currently a bleak diagnosis and prognosis, and responsive social care services would be needed to meet social, domestic and occupational care needs. However, there is a workforce crisis in social care in countries like the United Kingdom (Dromey & Hochlaf, 2018), which the Covid‐19 pandemic has severely exacerbated (Pearson et al., 2022). There is a real risk of sleepwalking into the roll out of “exciting” new technological screening or detection opportunities without proper planning and resourcing of mental health and social care services to receive the increase in workload that these technological innovations generate.

### 4.1 Strengths and limitations

Our approach to this review was strengthened by use of screening framework, this helped us to identify important ethical domains to consider when a new screening or early detection paradigm is emerging. However, a limitation is that our review has not followed systematic reviewing practice, we have covered too many domains for a single search string to pick up the range of literature which was needed to cover the issues in this field.

### 4.2 Next steps and final conclusions

We note a number of future activities in the field of dementia early detection which would be valuable in progressing this field in an ethical way. First, involving diverse patients, carers and clinicians in the development of these technologies would ensure that progress in this domain is acceptable and meets a patient or clinician need. It would also make it more likely that technologies will fit with clinician workflow and/or produce data which is valuable for, and interpretable by, clinicians (Ford, Edelman, et al., 2021). Second, it would benefit implementation of technologies if it is clear from the outset who these technologies are targeting. When considering the potential audience, issues of bias and inequality can then be evaluated. Developers should have clear strategies to prevent widening health inequalities via digital inequity.

Third, it must be clear to users of new technologies what benefits, if any, they can expect from using the technology. At a time where there is no disease‐modifying treatment for dementia, the potential for changing the course of their condition is small, and supportive care for dementia is often poorly resourced. For users to make an informed decision to use the technology, they should be clear on what the outcome may (or may not) lead to. It may also be preferable to regulate companies providing these digital tools, ensuring that they provide appropriate follow up for their clients who are flagged as having possible dementia.

Finally, we hope to have shown that frameworks for population screening are both relevant and useful for considering the ethical and practical challenges associated with digital technologies for early disease detection. While discussions of these challenges often focus on the process of diagnosis within a clinical setting, this wider population health approach can offer a valuable, tried and tested framework for those involved in developing and implementing the tools discussed here.

We summarize our recommendations for ethical practice in this field in Box 1.

BOX 1 **Recommendations for ethical practice in early detection of dementia**

*The early detection of dementia using widely available digital devices and automated systems should be considered as a potential form of population screening and those developing such tools should consider the relevance of ethical screening criteria*.

Early detection of dementia may identify a large number of people with mild cognitive impairment for whom it is not currently possible to determine if they will progress to dementia. Early detection models should ideally focus on accurate prediction of who will progress to dementia from MCI.

It is not ethical to implement population level early detection of dementia until:

1. There is a treatment available which changes the course of the disease and which is more effective when started earlier in the disease course;
2. We can accurately determine how likely someone is to progress to dementia in the prodromal or MCI phase;
3. There is a highly accurate detection method which is minimally invasive and cost‐effective;
4. An appropriately resourced health service pathway supports early detection, including (i) clinician training in using the detection method and communicating results, and (ii) diagnostic, psychological support and treatment services resourced to meet the additional need generated by the early detection program.

In addition, the following principles should be followed (cf. Arribas‐Ayllon, 2011):

5 The decision to undertake early detection of dementia should be voluntary on the part of the patient, free of coercion and based on informed consent; and the result should be kept confidential.
6 The potential for psycho‐social harm to the patient which could arise as a result of early detection should be assessed in each case.
7 Any deployment of early detection methods should be monitored for fairness, so that the method does not contribute to health inequalities.

### 4.3 Final conclusions

The evidence‐base for benefits from early detection of dementia using digital biomarkers and automated methods is weak, and the possibility of harm is tangible. Before these technologies become common place, it should be clear how newly detected patients with dementia can and should be supported and how technologies can be rolled out without exacerbating digital health inequalities.

## AUTHOR CONTRIBUTIONS

**Elizabeth Ford:** Conceptualization (equal); formal analysis (equal); project administration (equal); writing – original draft (equal). **Richard Milne:** Conceptualization (supporting); writing – original draft (supporting); writing – review and editing (equal). **Keegan Curlewis:** Data curation (supporting); investigation (supporting); writing – review and editing (supporting).

## FUNDING INFORMATION

Elizabeth Ford acknowledges funding from the NIHR Applied Research Collaboration Kent Surrey and Sussex (grant number NIHR200179). Views expressed are those of the authors and not necessarily those of the NIHR or the Department of Health and Social Care. Richard Milne acknowledges funding from the Wellcome Trust (grant number 108413/A/15/D).

## CONFLICT OF INTEREST STATEMENT

The authors have declared no conflicts of interest for this article.

## RELATED WIREs ARTICLES

Computational resources in healthcare

Integrative biomarker discovery in neurodegenerative diseases

## Data Availability

Data sharing is not applicable to this article as no new data were created or analyzed in this study.
